# Colonyzer: automated quantification of micro-organism growth characteristics on solid agar

**DOI:** 10.1186/1471-2105-11-287

**Published:** 2010-05-28

**Authors:** Conor Lawless, Darren J Wilkinson, Alexander Young, Stephen G Addinall, David A Lydall

**Affiliations:** 1 Centre for Integrated Systems Biology of Ageing and Nutrition, Institute for Ageing and Health, Newcastle University, Campus for Ageing and Vitality, Newcastle-Upon-Tyne, NE4 5PL, UK; 2 School of Mathematics & Statistics, Herschel Building, Newcastle University, Newcastle-Upon-Tyne, NE1 7RU, UK

## Abstract

**Background:**

High-throughput screens comparing growth rates of arrays of distinct micro-organism cultures on solid agar are useful, rapid methods of quantifying genetic interactions. Growth rate is an informative phenotype which can be estimated by measuring cell densities at one or more times after inoculation. Precise estimates can be made by inoculating cultures onto agar and capturing cell density frequently by plate-scanning or photography, especially throughout the exponential growth phase, and summarising growth with a simple dynamic model (e.g. the logistic growth model). In order to parametrize such a model, a robust image analysis tool capable of capturing a wide range of cell densities from plate photographs is required.

**Results:**

Colonyzer is a collection of image analysis algorithms for automatic quantification of the size, granularity, colour and location of micro-organism cultures grown on solid agar. Colonyzer is uniquely sensitive to extremely low cell densities photographed after dilute liquid culture inoculation (spotting) due to image segmentation using a mixed Gaussian model for plate-wide thresholding based on pixel intensity. Colonyzer is robust to slight experimental imperfections and corrects for lighting gradients which would otherwise introduce spatial bias to cell density estimates without the need for imaging dummy plates. Colonyzer is general enough to quantify cultures growing in any rectangular array format, either growing after pinning with a dense inoculum or growing with the irregular morphology characteristic of spotted cultures. Colonyzer was developed using the open source packages: Python, RPy and the Python Imaging Library and its source code and documentation are available on SourceForge under GNU General Public License. Colonyzer is adaptable to suit specific requirements: e.g. automatic detection of cultures at irregular locations on streaked plates for robotic picking, or decreasing analysis time by disabling components such as lighting correction or colour measures.

**Conclusion:**

Colonyzer can automatically quantify culture growth from large batches of captured images of microbial cultures grown during genome-wide scans over the wide range of cell densities observable after highly dilute liquid spot inoculation, as well as after more concentrated pinning inoculation. Colonyzer is open-source, allowing users to assess it, adapt it to particular research requirements and to contribute to its development.

## Background

**Figure 1:**
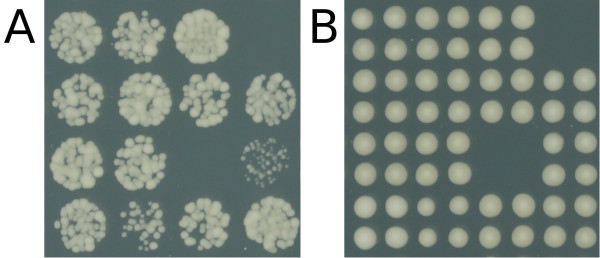
**Cultures can be spotted or pinned onto agar**. A) 16 different *S. cerevisiae *mutant cultures growing in 384-format on solid agar after inoculation by spotting of dilute liquid inoculum. B) The same 16 mutants, growing in quadruplicate cultures on a different plate in the same location, in 1536-format , inoculated by direct pinning onto agar. Images were simultaneously captured 2 days after inoculation. Mutant specific differences in cell densities are apparent in both images, but more pronounced in panel A.

Spotted cultures grown on solid agar are commonly used by microbiologists as "spot tests" where serially diluted cultures are inoculated, typically by hand, onto agar plates, and resultant differences in growth over the range of dilutions are used to distinguish between the fitness of mutants. Performing high-throughput screening of arrays of genetically distinct micro-organism cultures growing on solid agar plates is a useful way to screen for genetic interactions or for the effects of small-molecules (e.g. drugs) or environment (e.g. temperature or nutrient availability) on organisms of different genotypes using this method. This is particularly true for model organisms such as *Saccharomyces cerevisiae *(brewer's yeast). Growing cultures on solid agar has some advantages over growth in liquid medium including speed and ease of robotic handling. To grow microbe cultures on solid agar, cultures are inoculated by direct pinning onto solid agar plates, or inoculated from liquid after dilution (spotting). Liquid spotted culture inoculum can be of arbitrary cell density. The inoculation method used affects the initial cell density for each culture on the plate, and the resulting final achievable densities (see Fig. 1). Cell populations grow at a rate depending on the viability of their particular genotype in the plate environment (e.g. depending on agar nutrient availability, the presence or absence of drugs in the agar or temperature). Observed differences in growth rates reveal which genotypes grow best, if at all, in a given environment, allowing conclusions to be drawn about gene function. Large, qualitative differences in growth can easily be observed by eye (as in Addinall et al. [1]), but quantitative measures of growth are required to reliably detect small differences in a high-throughput context and to demonstrate the statistical significance of differences.

In order to quantify data produced using this technique, we decided to develop Colonyzer, a new tool for generating several measures of culture cell density and morphology including area, integrated optical density (IOD), colour and granularity which emphasises being able to reliably detect cultures with low cell densities. These measures are appropriate for addressing a wide range of research questions and can be disabled or ignored by the user as required. Colonyzer is capable of detecting low cell densities after performing background lighting correction to allow a fair comparison between the densities of all cell cultures on a plate and implementing a Gaussian mixed model image segmentation algorithm which robustly detects the location of cultures barely visible to the human eye.

Culture density can be estimated from agar plate images, either captured as photographs or by plate-scanning, by segmenting images of growth into agar and culture areas. There are several tools currently available which can perform this segmentation, for example *HT Colony Grid Analyzer *described by Collins et al. [2], *YeastXtract *as described by Shah et al. [3], the proprietary *Colony Imager *software distributed by S&P robotics [4] (Toronto, Canada), or the multi-purpose *CellProfiler *described by Lamprecht et al. [5] However these tools have some drawbacks, particularly for spotted cultures, as summarised in Table 1. The performances of *CellProfiler *and *HT Colony Analyzer *are directly compared with that of Colonyzer in the Results & Discussion section.

**Table 1:** Summary of image analysis tool features.

	Spotted Cultures	Pinned Cultures	Lighting Correction	Fast	Open Source	Simple Interface	Colony IOD	**Non-Comm. Dev**.
**Colonyzer**	•	•	•		•	•	•	•
**HT Colony Analyzer**		•		•	•	•		•
**YeastXtract**	•	•		•	?	?	?	•
**Colony Imager**		•		•		•		**N/A**
**CellProfiler**	•	•	•	•	•		•	

Colony Integrated Optical Density (IOD) is a measure of cell density appropriate for spotted cultures, calculated as the sum of pixel intensities in an area found to contain cells. The Non-Comm. Dev. column indicates whether code development and assessment requires no commercial licenses (such as Matlab). *YeastXtract *code and executable has not been available for download at its published web address for the past several months.

Most image analysis tools for quantifying the growth of gridded colonies do not deal well with the irregular spot morphology which results from highly dilute inoculation of liquid spots (e.g. Fig. 1A). This appears to be because tools of this kind are primarily designed to quantify final cultures with high cell densities which have been either pinned onto agar or spotted with highly concentrate inoculum. These particular inoculation techniques give rise to clearly defined, opaque, circular cultures (e.g. Fig. 1B).

**Figure 2:**
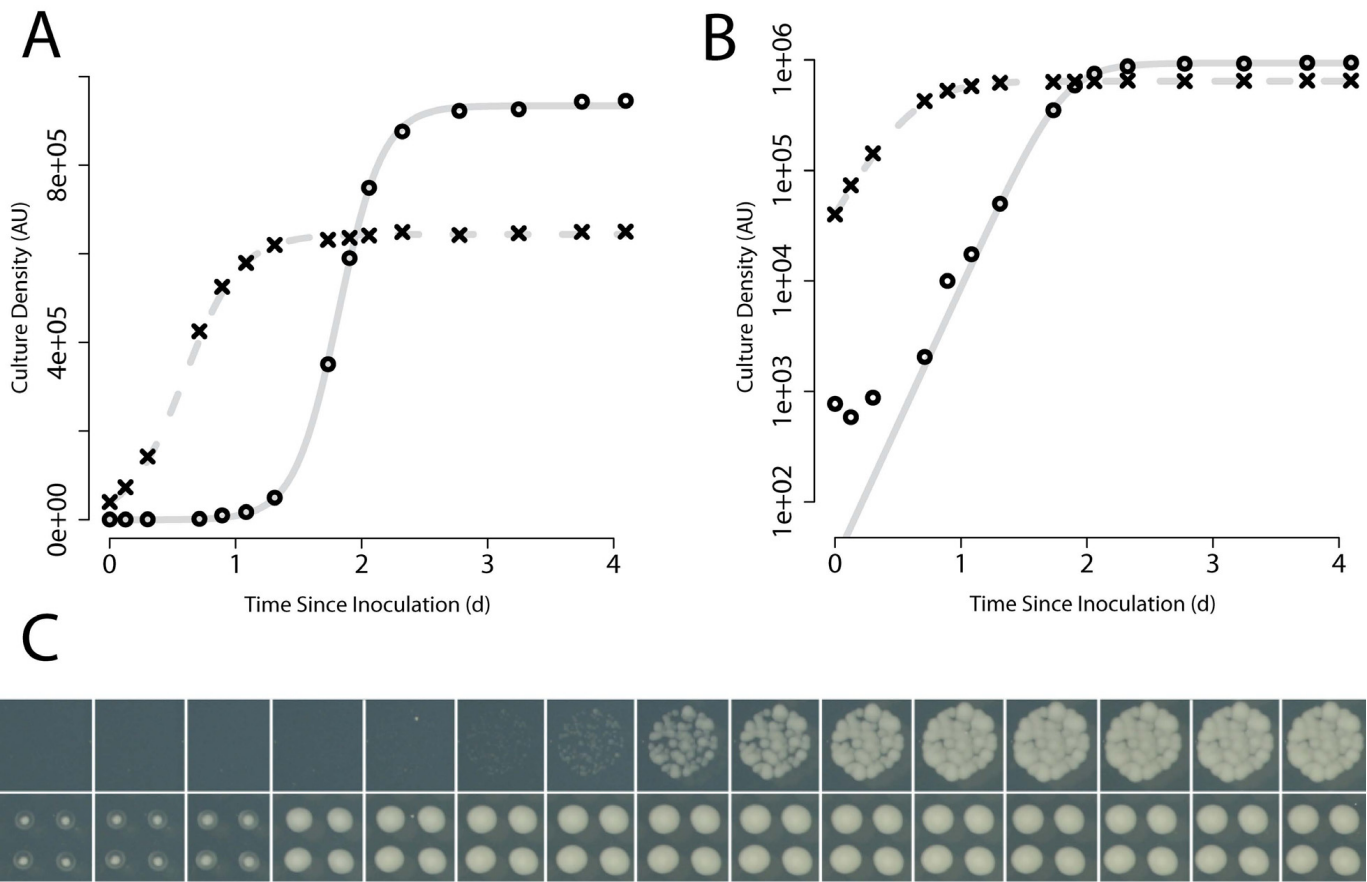
**Logistic growth model fit**. A) & B) Logistic model (grey curves) fit to a timecourse of cell density observations (black symbols) for identical *S. cerevisiae *mutants grown from a dilute liquid inoculated (spotted) culture on solid agar in 384 spot format (open black circles) and from direct pin inoculation (black crosses) in 1536 format at 23°C. Cell density is estimated from IOD using Colonyzer. The logistic model is fully described by the three parameters: *G(0)*, the inoculum cell density, *K *the carrying capacity or maximum achievable cell density for that culture and *r *the culture growth rate (d^-1^). Images were captured manually, in parallel for both inoculation types. A) Observations and model estimates plotted with density on the linear scale. B) Observations and model estimates plotted with density on the log scale. C) Culture photograph timecourses in 384 spotted and 1536 pinned formats corresponding to the data in A) & B). The lower timecourse (1536) format has pinned cultures in quadruplicate. The sum of these four culture densities is represented by the black crosses in panels A) & B).

There are two advantages to using dilute, liquid inoculum in these high-throughput screens: 1) The wider range of cell densities observed during the growth period can be used to more accurately parametrise dynamic simulation models of cell population growth, and is likely to be more sensitive to differences between phenotypes. The longer growth period observed in spotted cultures affords more experimental opportunity for image capture over a wider range of growth. This is important for characterising growth kinetics since information about model parameter values can best be derived from data where cell density is changing most rapidly. 2) Micro-colony formation and growth can be observed microscopically in independent, spatially distinct colonies of poorly growing cultures which may only go through a few divisions before growth arrest. This allows direct, detailed follow up of cultures flagged as poor growers by high-throughput analysis (e.g. the culture on row 3 column 4 in Fig. 1A). For a range of examples of liquid inoculated spots, see the upper timecourse in Fig. 2C.

We can observe the different window of opportunity for capturing changes in cell density in spotted and pinned cultures in Fig. 2. The pinned cultures grow from inoculum to carrying capacity in one day, whereas the same cultures growing from the more dilute spotting inoculum grow from inoculum to carrying capacity in over two days. Given practical limitations to the frequency of image capture, particularly overnight, spotted cultures will give more information about cell division rates than pinned cultures. Shah et al. [3] demonstrate the utility of the logistic population model for summarising the growth of *S. cerevisiae *cultures grown on agar, captured as scanned plate images, and this same approach is demonstrated in Fig. 2 using Colonyzer output. Estimated, maximum (exponential-phase) growth rates may be more directly related to the phenotype of interest than final cell density, which likely depends on competition between distinct cultures for space and nutrients and slight variation in inoculum concentrations for instance, as well as on intrinsic growth rates resulting from culture genotype. In order to take full advantage of summarising the wide range of cell density observations produced in a timecourse experiment with a dynamic model, it is important to be able to quantify very low, early, exponential-phase cell densities.

In order to detect low cell densities by photography it is necessary to correct for any lighting gradients which exist across the image after the image is captured. Even in professionally designed, purpose-built plate photographing systems (such as *S&P Robotics SPImager *and *BM3-SC *[4]), we have found significant lighting gradients in captured images. We have found that the best strategy to overcome this is to detect any existing gradients after image capture and to compensate for them computationally. Culture IOD (the sum of pixel intensities over an area of an image which is classified as belonging to an individual culture) is an alternative to culture area as a measure of cell density. In dilute *S. cerevisiae *cultures, for example, at low densities, cultures are opaque and so close to the colour of the agar. As cell density increases, cultures become thicker and begin to take on a colour characteristic of the micro-organism, which is generally different to that of the agar. This colour difference provides a rough measure of culture thickness. Opacity varies in a non-linear fashion with colony thickness over some thickness range and so IOD is an imperfect surrogate for cell density, however, we have observed that lighting-corrected IOD dynamics are less noisy, and a more perfect fit to the logistic model than direct area data. Without lighting correction, the IOD of cultures which happen to be in brightly lit locations on the plate is overestimated. Overall, the observation that culture opacity changes with cell density and our ability to correct for lighting gradients which would otherwise bias density estimates suggests that lighting-corrected IOD should be a better estimator of cell density than culture area alone.

**Figure 3:**
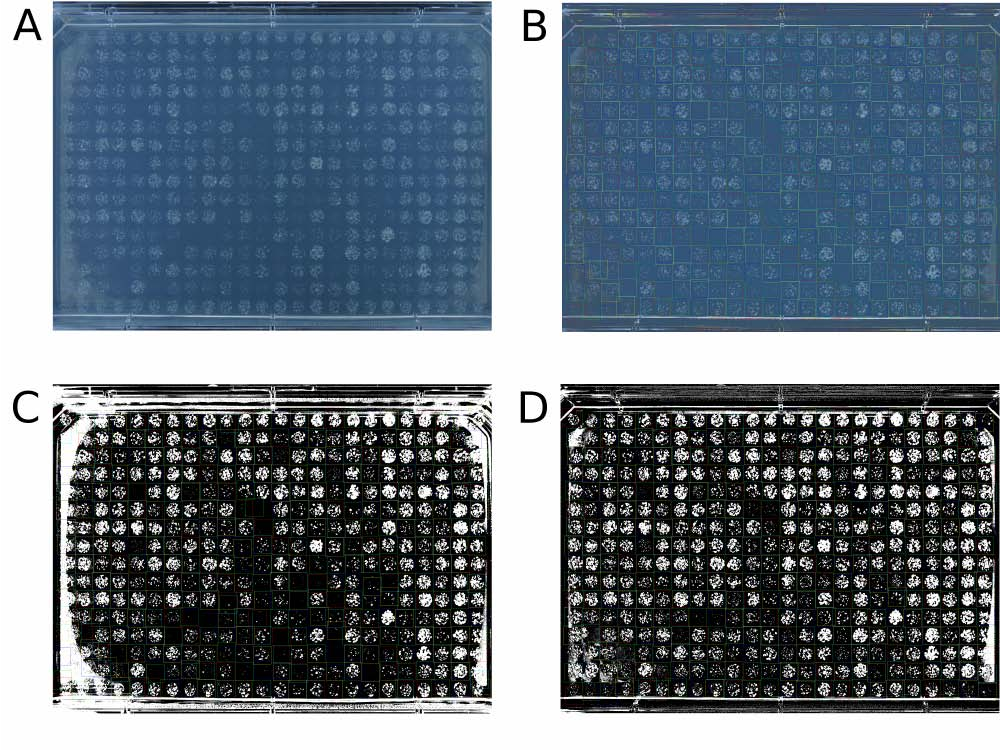
**Colonyzer lighting correction & thresholding**. Colonyzer detects cultures with low cell density in liquid-inoculated spots in 384 format. A) Original captured plate image from S&P Robotics [4] BM3-SC, B) Image after lighting correction, C) Thresholded image without prior lighting correction, D) Thresholded image with prior lighting correction. Coloured squares around cultures are tile locations as estimated by Colonyzer. In panels C) & D), white pixels are those classified as being cell cultures, black pixels are classified as being agar. Pixel misclassification on some non-experimental edge colonies in panel D) are artefacts caused by light reflecting from plate walls onto the agar. Colonyzer largely (but not completely) corrects for these artefacts when they occur.

In order to calculate culture area or IOD, image analysis tools must first classify pixels as belonging to culture or agar. One way to do this is to classify pixels by intensity (or brightness) thresholding. Again we find that the intensity difference between cultures with low cell density and the background agar can be less than the difference in pixel intensities throughout the plate caused by lighting effects. The result is that, without lighting gradient correction, in darker parts of the image, poorly growing or low cell density cultures are more likely to be classified as background agar than in brighter parts, introducing a spatial bias in detection of poorly growing cultures or early growth. Comparing Fig. 3C &3D we can observe the spatial bias which can occur without lighting correction.

## Implementation

### Algorithm Overview

**Figure 4:**
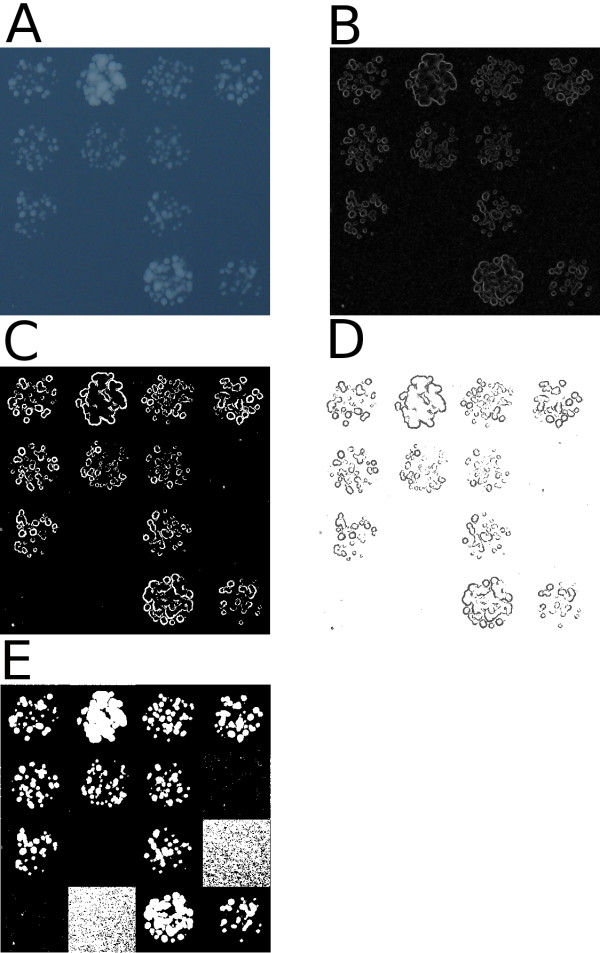
**Local thresholding algorithm for fast, sensitive first-pass image segmentation**. A) 16 spotted cultures from an example original plate image, B) Sobel gradient map of panel A, C) Mask of top 5% intensity gradients from panel B, D) Mask applied to original image (background set to white) E) Original image intensities locally thresholded by tile to exclude the darkest 33% of pixels from panel D in each tile location

**Figure 5:**
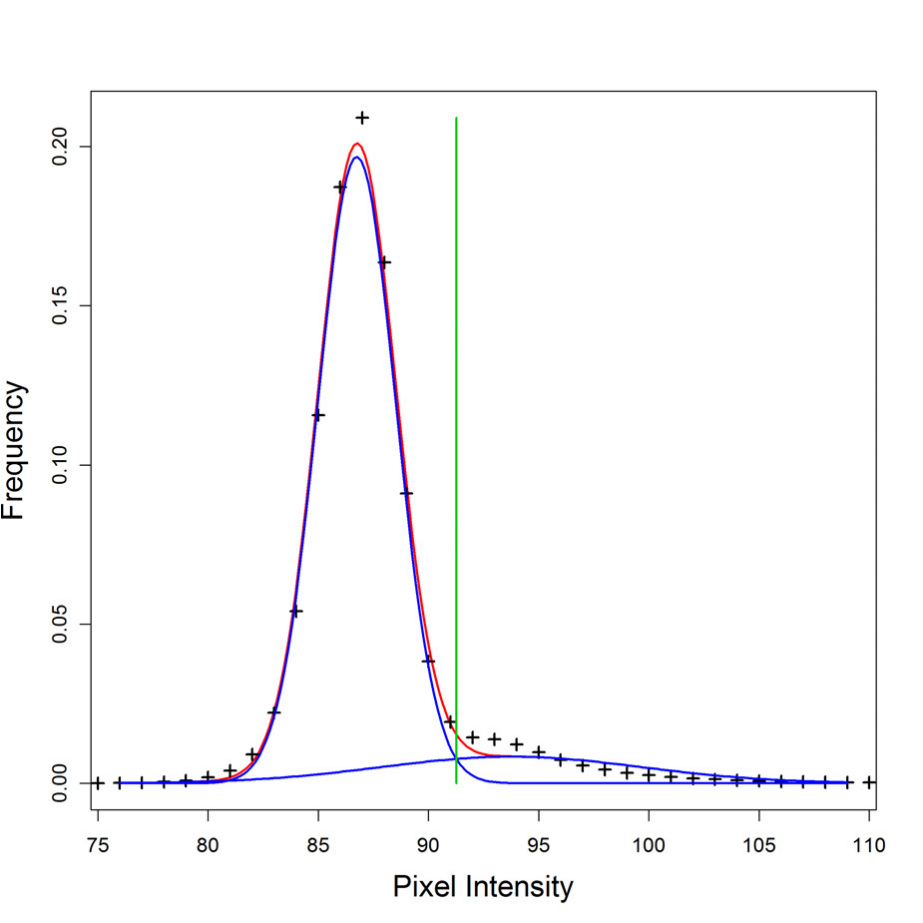
**Automatic Thresholding with Gaussian mixed model**. Example trimmed image pixel intensity distribution (black crosses), maximum likelihood model estimate for mixed model (red curve), individual Gaussian components modelling agar and culture intensities separately (blue curves) and optimal threshold *x*_*Thresh *_(green line) at intersection of the two component distributions. This example histogram represents a plate image with a strong agar signal and a low cell density (culture barely detectable by eye).

The user captures a photograph of an array of cultures on a plate (typically in .jpg format) at one or more times after inoculation, by photographing or scanning the plate. For high-throughput screens, inoculation and image capture are typically carried out with robotic assistance, and photographic image capture can sometimes be carried out more frequently and cheaply than scanning. The user provides an estimate of the location of cultures on the plate (typically arrayed on a rectangular grid in 48, 96, 384, 768 or 1536 format). Colonyzer segments the image locally by thresholding to capture the steepest intensity gradients in an area of the image, roughly classifying cultures and agar (see Fig. 4). Colonyzer then creates a pseudo-empty plate image by replacing locally thresholded culture pixels with appropriate interpolated background pixels. This image is used to create a background lighting map to adjust for spatial lighting gradients: pixels in the pseudo-empty image are regressed towards the median background intensity on the plate, and the same regression is applied to the original image. Finally the corrected image is segmented by maximising the log-likelihood of a two-component Gaussian mixed model, and thresholding at a pixel intensity which has an equal probability of being classified as culture or agar (see Fig. 5). This allows plate-wide, gradient independent segmentation capable of detecting cultures of extremely low cell density where culture and agar intensities are similar, due to culture opacity. Segmentation allows Colonyzer to locate irregularly shaped cultures (typical of dilute liquid inoculum for example) precisely in space, before estimating their area, integrated optical density, colour and granularity.

Quantification of captured timecourses of the growth of spotted culture cell densities arrayed on 384-spot format solid agar plates, from photographs while correcting for an existing lighting gradient is the most difficult scenario for an image analysis tool for quantifying culture growth. Colonyzer can perform this task, and its algorithms are also suitable for the quantification of simpler images (e.g. quantifying pinned culture size). Colonyzer is therefore general enough to be suitable for all solid agar culture density analyses.

Colonyzer is a mixture of algorithms, some of which are original, which have been integrated as a Python [6] script using the statistical package R [7] (via the Python library RPy [8]) with the R package rgenoud [9] and uses the Python Imaging Library [10] and Numerical Python [11] extensively. It is available for download under GNU general public license [12]. Despite the fact that Colonyzer has been optimised for sensitivity, generality and robustness rather than speed, deploying these algorithms on a Linux cluster of 90 CPUs, we can reliably quantify cell density for photographs representing over 2 million cultures (photographed repeatedly in timecourse experiments) arrayed in 384-format overnight.

### User-input

This method starts with an image of a relevant plate. Colonyzer is currently designed to work with a rectangular grid of cultures (with edges parallel to the image), but could easily be adapted to work with arbitrarily placed cultures. To help Colonyzer locate the cultures on a rectangular grid, the horizontal (*x_dim_*) and vertical (*y_dim_*) dimensions (pixels) of a typical rectangle completely containing a culture (a tile) are estimated along with the coordinates (*x_start_*, *y_start_*) (pixels) of the top left hand corner of the top left hand tile (see Eq. 1) The user provides the number of tile rows (*N*_*rows*_) and columns (*N*_*cols*_) on the grid, together with estimates of the locations of the centres of the top left and bottom right spots (*x*_*tl*_, *y*_*tl *_and *b*_*br*_, *y*_*br *_respectively). The algorithm begins with an estimated tile location of  (*x_start_*, *y_start_*) and steps *x_dim_* horizontally and *y_dim_* vertically Ncols and Nrows times respectively to provide starting guesses for optimal tile locations. These guesses are then improved on a tile-by-tile basis by brute force minimisation of the number of pixels classified as culture which are present on the edge of the tile. These estimates become critical for smaller culture sizes (e.g. cultures arrayed in 1536 format), and so we have developed a GUI tool called Parametryzer [12] for rapid estimation of spot locations for batches of images.(1)

Having provided these estimates once for an entire batch, or on an image-by-image basis as appropriate, the user simply executes the Colonyzer script, which runs through (usually large) lists of images stored in a given directory, and generates textual output files appropriate for further analysis. No further user input is required.

### Correction of spatial lighting gradient

**Figure 6:**
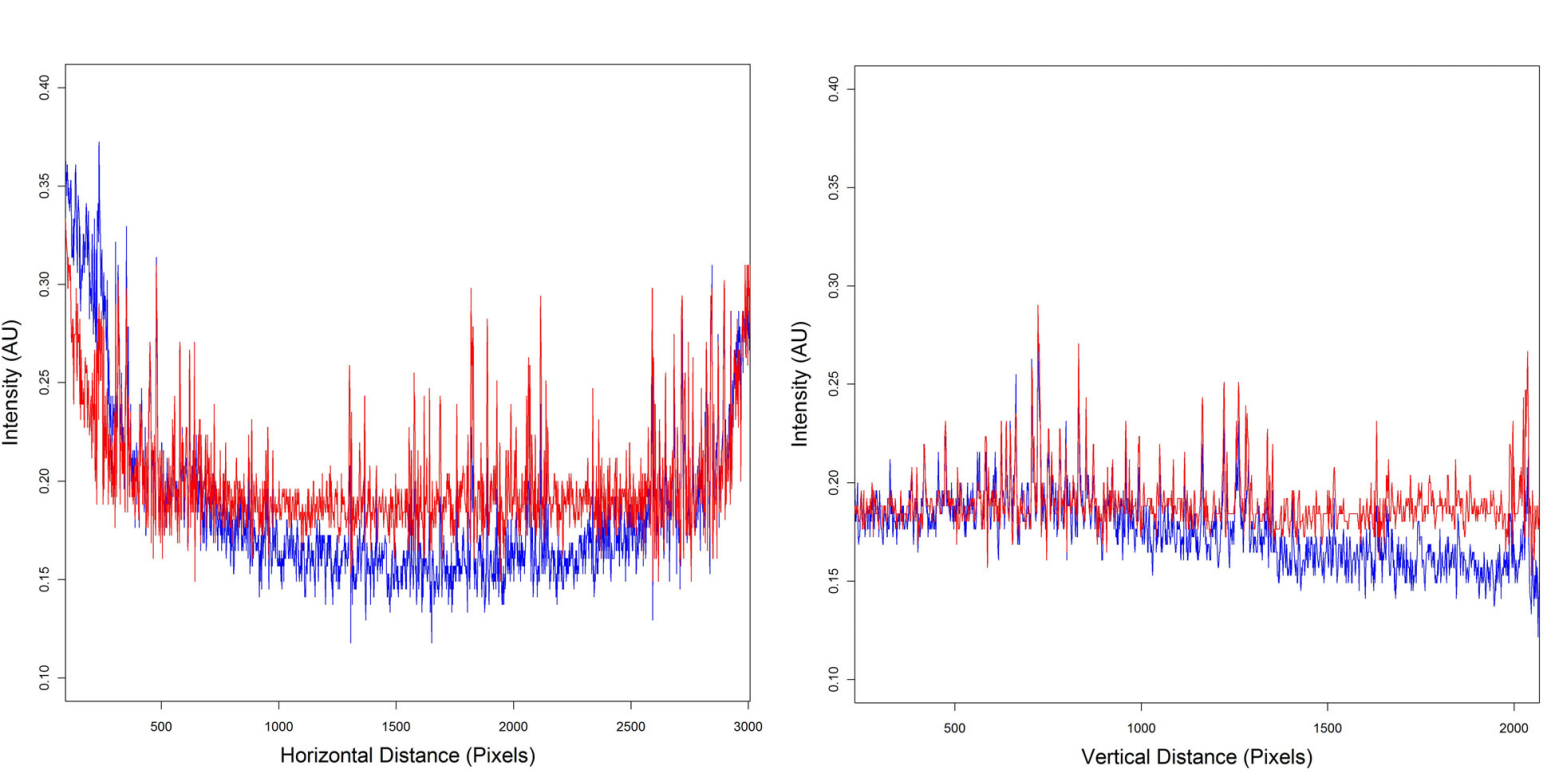
**Colonyzer corrects spatial lighting gradients**. Horizontal and vertical intensity slices from a 384 well plate image with growing S. cerevisiae spots captured on an S&P robotics [4] BM3-SC. Local intensity peaks represent cultures (at low cell density) or plate edges. Blue curves show the lighting-induced intensity gradient before correction. Red curves show a flat intensity gradient after correction, which maintains culture information.

To counter the effects of lighting gradients, Colonyzer constructs a lighting map for the whole image by building a pseudo-empty plate, which is the best estimate of what that particular agar plate alone (with no cultures) would look like. Then a correction map is created which would regress all pixels on that pseudo-empty plate back towards the median agar intensity. Finally that correction map is applied to the original image to remove all lighting bias. A pseudo-empty agar plate is most appropriate for this analysis as there are often reflective artefacts and halos surrounding the cultures which are caused by light reflecting from the sides of the petri dish and from culture reflections through the translucent agar on which they are growing (see Fig. 3A for example). Constructing a pseudo-empty plate instead of using, say, a neutral grey sheet has the added advantage of partially eliminating these unwanted artefacts while correcting the gradient (e.g Fig 3B &3D). Fig. 3 demonstrates the effect of lighting gradients on the segmentation of cultures with low cell density. Comparing Fig. 3C &3D we can see that, without correction, many cultures have incorrectly been allocated zero growth estimates, particularly in the bottom centre of the sample plate. We can see that in this example image, glare on some of the edge colonies, from the plastic walls of the plate has caused Colonyzer to overestimate their area in both cases (although to a lesser extent after correction). This edge effect can be seen to remain after lighting correction in Fig. 6. We have not attempted to completely eradicate this glare problem since edge cultures on 384-format plates should be non-experimental. These cultures have a competitive advantage over spots in the middle of the plate since they have access to greater amounts of nutrients, therefore comparative analysis of their density is always misleading. This does not seem to be the case for less dense formats like 96-format, however.

Pseudo-empty plates are constructed by cutting culture pixels out of the plate image and filling in the remaining gaps with interpolated background agar intensities. Culture pixels are cut out using a sensitive thresholding algorithm based on morphological edge detection to segment the image into agar and culture, similar to that presented by Chen et al. [13], on a tile-by-tile basis. The original image is then masked, cutting out culture areas, leaving only agar behind.

#### First-pass local thresholding algorithm

▪ Take the original RGB image (Fig. 4A), and convert it to greyscale.

▪ Create an intensity gradient map using the Sobel algorithm [14] (Fig. 4B)

▪ Generate a gradient histogram for a user-estimated tile location. Estimate the gradient value below which 95% of the pixels in the tile lies, and threshold the gradient map for the tile at this gradient intensity (Fig. 4C).

▪ Use this binary image as a mask on the greyscale image (Fig. 4D). In this way the pixels whose intensity gradient is in the top 5% for the colonies are cut out (the edges have been masked).

▪ For each approximate rectangular tile location, as estimated from the user input, that tile is cut out of the masked image. The intensity histogram for that masked tile is constructed and the original image tile thresholded so that the darkest 33% of the masked pixels are allocated as background (Fig. 4E).

The 5% and 33% cutoff frequencies were chosen heuristically to suit the wide range of images we have analysed but essentially they are cutting out the very highest gradients, and then thresholding to exclude the darkest pixels having those high gradients. We implemented this particular method of image segmentation since it is very fast (important for high-throughput analysis) and it locally adapts its sensitivity depending on the amount of signal present. Signal in this case is the intensity of growing cultures, which depends on cell density in each tile (which can vary across tiles within a plate), but also on the light incident on a particular colony. We simply require that any signal is correctly identified as culture. Misclassification of agar pixels as culture is not important since misclassified pixels will be cut out and filled in with an approximation of agar intensity in the next step. The opposite, misclassification of culture pixels as agar, would lead to significant errors as our algorithm would later "correct" the intensity of the culture towards the background median during the gradient correction, thereby eradicating the intensity signal for these pixels. It would be trivial to adapt this method to threshold on a pixel-by-pixel basis (using user defined tile dimensions), rather then on a tile-by-tile basis as presented here, however the tile thresholding step would be *~O(x*_dim_*y_*dim*_) times slower.

### Creation of a pseudo-empty plate

There are two slight practical problems with cutting out the culture pixels and replacing them with interpolated background pixels. Firstly, any segmentation algorithm will classify the very edges of a culture as background, since including all of the culture would likely mean classifying much background noise as culture. Secondly, depending on the image capture and lighting methods used, there can be reflective haloes surrounding cultures from reflections through the translucent agar medium and off the surface beneath the plate, as well as reflective glare from the sides of the petri dishes. Both of these phenomena imply that immediately outside the thresholded area, a strip of pixels which are of higher intensity than the true background agar intensity is often seen. To resolve this, we search from the edge of a thresholded culture area over a distance approximately equal to the radius of a typical culture (e.g. *x*_*dim*_/2) to find the darkest pixel in that range and use that as the edge intensity for filling in the gaps.

#### Pseudo-empty plate construction

▪ Strongly smooth the greyscale version of the original image. This averages out the background values and reduces the chance of extreme values being selected for interpolation.

▪ For each image row, scan horizontally identifying gap edges from the thresholded map.

▪ At each edge, search away from the gap for a distance of *x*_*dim*_/2 pixels and take the darkest pixel in that region. Use this as the background edge value.

▪ Step across the gap on the same row and find the edge on the other side, repeat the previous step to get a background edge value for this side of the gap.

▪ Now fill in the gap-slice by linearly interpolating between the two newly found edge values

▪ Create a new smoothed copy of the original greyscale image.

▪ Repeat this process, but this time, scan vertically (along image columns instead, and using a search distance of *y*_*dim*_/2)

▪ Merge the vertically-scanned and horizontally-scanned images by taking the minimum value at each pixel location. This is the pseudo-empty plate image

Scanning horizontally and then vertically, taking the minimum pixel value for the two scans is necessary since the irregular morphology of some cultures occasionally results in islands of bright pixels within a defined gap. This results in one edge being brighter than intended, affecting the interpolation. This technique reduces the chance of a given gap pixel being filled with an excessively bright intensity.

### Regression towards median background intensity

From the smoothed, masked greyscale image, the user-estimated area containing growing spots is cut out and its median background pixel intensity calculated. Then, for each pixel on the pseudo-empty plate, the ratio *R*_*CORR *_is calculated: *R*_*CORR *_= *I*_*MED*_/*I*_*PE *_where *I*_*PE *_is the pseudo-empty pixel intensity and *I*_*MED *_is the median background pixel intensity for the plate. On a per-pixel basis the pixel intensity of the original image (*I*_*ORIG*_) is scaled so that the new intensity is *R*_CORR_*I_*ORIG *_in order to correct the lighting gradient on the plate. For non-saturated source images the resulting corrected image maintains the signal of interest, as demonstrated in Fig. 6.

### Segmentation algorithm

Colonyzer segments the lighting-corrected image into agar and culture areas, using a method similar to that presented by Huang and Chau [15]. A mixed model of two Gaussian distributions (*g*, Eq. 2) is constructed which models the histogram of pixel intensities for the entire corrected image (*μ*_*k *_is the expected value of Gaussian *k*, *σ*_*k *_is the standard deviation of Gaussian *k *and *θ *is the ratio between the peak heights of the two Gaussian curves representing agar pixels and culture pixels):(2)

By constraining the number of Gaussian components in the mixed model to exactly two, we insist that all pixels be classified as either culture or agar. Parameter estimation is carried out by constructing a log likelihood estimator for the model *g*, given the pixel intensity histogram *PI[0:255] *(Eq. 3) and maximising it with respect to its parameters (Eq. 4).(3)

The optimum parameters (denoted by *) are estimated as follows using the genetic optimisation package rgenoud [9]. Fig. 5 shows an example of the fit.(4)

The most appropriate threshold intensity is then estimated by numerically solving the following for intensity *x*_*thresh *_and selecting the root which gives the largest value for (5)

Huang and Chau [15] segment images by taking the mean intensities of the Gaussian components and using the average of these mixture means as their threshold. This assumes that each segment contains a roughly equal proportion of the image pixels. For this particular problem, the intersection of the two component distributions with the highest intensity is the most appropriate threshold since this is the intensity at which the probability of a pixel being assigned to either foreground or background is identical. Intensities either side of the intersection threshold are on balance of probabilities most likely to belong to culture or agar. Performing the final image segmentation with this mixed model approach allows for extremely sensitive segmentation in conditions of low growth. Clear spot signal (which has been maintained through the lighting correction algorithm) can be picked up where only agar is visible with the naked eye.

### Location of colony tiles

In an ideal experiment, cultures would remain entirely within the approximately square tiles that they were intended to be inoculated into on the rectangular agar grid. Biological heterogeneity, overgrowth or slight errors in plate location or alignment during image capture necessitate improving on initial location guesses before quantifying cell density. Colonyzer fine-tunes tile location estimates by taking the corrected, thresholded image, and for each tile, minimizing (by brute-force optimization) the number of culture pixels on the tile edge by changing the tile location while keeping its size fixed. Colonyzer performs this search over a 20*20 pixel search area.

### Measurements

Once an exact tile location has been found, Colonyzer stores the coordinates of the top left hand corner of the tile together with its *x*_*dim *_and *y*_*dim*_, allowing users to access sub-images of individual tiles if required. Then the number of culture pixels in the thresholded image are counted and this is stored as a measure of culture area. Similarly, the number of pixels in the tile's location on the gradient map are counted as a measure of culture morphology (i.e. texture or granularity). The original image is masked with the thresholded tile and the mean colour (RGB triplet) of the culture, and mean background agar colour are calculated and stored. The lighting corrected image is masked with the thresholded tile and the sum of the culture intensities in the masked image (less the median background intensity) is used as a measure of IOD. The sum of all the pixel intensities in the tile (less the median background intensity) is calculated as another measure of cell density. All of these values are written to a tab-delimited text file, together with row and column number for subsequent analysis.

## Results and Discussion

**Figure 7:**
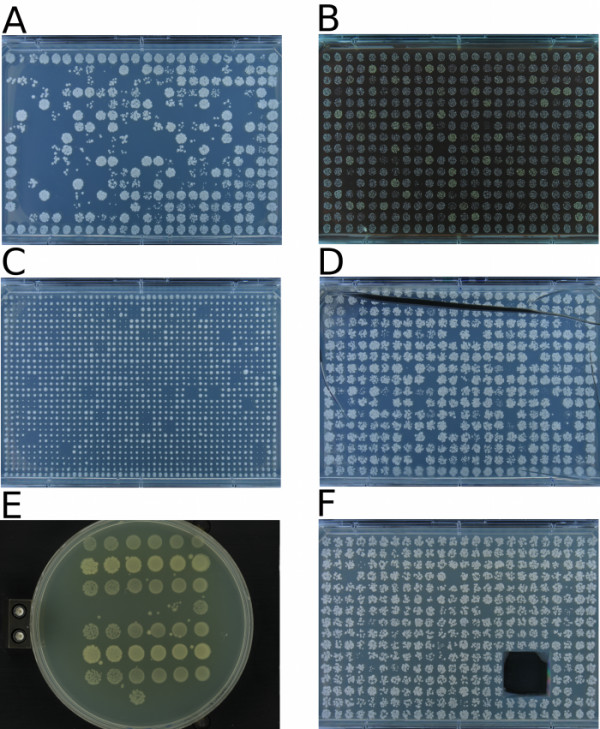
**Example Colonyzer input**. Some example photographs of *S. cerevisiae, S. pombe *and *E. Coli *cultures growing on solid agar with various spotting/pinning formats which are suitable for analysis using Colonyzer. A) Liquid inoculated 384 spot plate, B) 384 spot plate with a coloured drug in the agar, C) 1536 colony plate (pinned), D) 384 spot plate with an agar crack from drying E) Circular petri dish with serial dilution of rectangular gridded *E. coli *spotted cultures F) 384 spot plate with strongly growing contaminant cut out by hand to prevent overgrowth. Panels D and F are extremely rare worst-case scenarios for experimental plates and are only included to demonstrate that Colonyzer is robust to these features. All of these images (plus more examples) are available for download and testing [12].

Colonyzer is a tool for the quantification of the cell density in micro-organism cultures growing on solid agar from images of growth. It is sensitive enough to quantify extremely low cell densities in the presence of a spatial lighting gradient. We have demonstrated that information about cultures with low cell densities can be reliably extracted from plate photographs by implementing lighting correction (see Fig. 3 for example) and using a Gaussian mixed model of pixel intensity distribution for image segmentation. Colonyzer also estimates other measures of density and morphology, such as granularity, IOD and colour. It is general enough to also deal with the simpler problem of quantifying culture density of pinned or dense-inoculum liquid spots and is fully automated, requiring minimal user input. Colonyzer can quantify cultures of *Sacchromyces cerevisiae, Schizosacchromyces pombe *and *Escherichia coli *(Fig. 7). We can see from the 384-format timecourses in Fig. 2B and Fig. 2C that Colonyzer can reliably detect cultures with cell density so low that they are barely observable by eye (see from fourth datapoint onwards).

**Figure 8:**
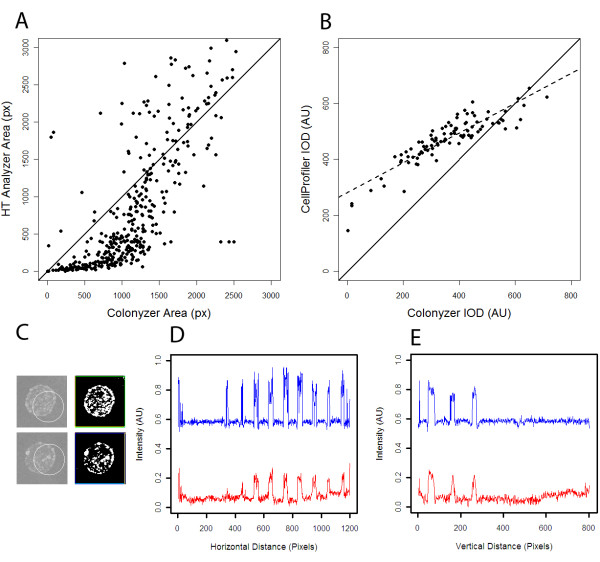
**Comparison of cell density estimates from different tools**. A) *HT Colony Analyzer *culture area estimate plotted against Colonyzer area estimate for the 384-format *Sample4.jpg *image from the Colonyzer example image collection hosted on SourceForge. Note that *HT Colony Analyzer *and several other high-throughput colony quantification tools are not designed to deal with the opacity and irregular morphology of spotted cultures and so this comparison is not completely fair. B) *CellProfiler *culture IOD estimate plotted against Colonyzer estimate for the 96-format yeast plate example from Figure 2E in Lamprecht et al. [5], downloaded from http://www.cellprofiler.org and analysed using the *CellProfiler *"Grid of Spots" demonstration pipeline. Pearson's correlation coefficient of 0.89, but with slope of 0.53. C) Two example cultures from the image in panel B with original images together with *CellProfiler *culture location (designated with a circle) in the left column. The right column shows the equivalent Colonyzer masks defining its estimates of cell locations *CellProfiler *incorrectly estimates culture location badly in three of the 96 cultures and erroneously trims ~5% of culture edge for almost all cultures. D) & E) Horizontal and vertical intensity slices through bottom and right sides of *CellProfiler *test image after correction by Colonlyzer (blue curve) and by *CellProfiler *(red curve).

We have compared the features of Colonyzer with those of several other software tools for quantifying culture density on solid agar (see Table 1 and Fig. 8). Most of these tools are designed to quantify pinned cultures where lighting correction and sensitive segmentation are not a critical issue. *HT Colony Analyzer *is one such tool, and Fig. 8A demonstrates quantitatively the large discrepancies between spot measurements made by this tool and the corresponding Colonyzer measurements. This is simply a reflection of the fact that *HT Colony Analyzer *(and other similar tools) are not designed to be able to quantify growing, dilute spotted culture cell densities. From Table 1 we can see that *CellProfiler *matches many of the features of Colonyzer. *CellProfiler *is a flexible image analysis environment, but its development has not been focussed on capturing growth kinetics of spotted cultures. Fig 8B demonstrates that there is much greater agreement between *CellProfiler *and Colonyzer density estimates than with simpler tools, but there are nevertheless some inconsistencies between them. The curvature apparent in the *CellProfiler *intensity profiles demonstrated in Fig 8D and 8E arises from incomplete lighting correction, particularly for cultures near the edge of the plate. The *CellProfiler* test image does not contain cultures of particularly low cell densities, but this degree of uncorrected intensity bias would cause bias in IOD estimates for low densities. *CellProfiler *also mis-identifies some example culture locations (Fig. 8C) which would lead to associated errors in IOD estimates. *CellProfiler *is significantly faster at performing this analysis than Colonyzer (~1 min and ~3 mins respectively on a modern workstation), however CPU time is relatively cheap and accuracy and high sensitivity for this particular image analysis problem were priorities during Colonyzer development.

To date we have used Colonyzer to quantify sets of timecourse photographs for several thousand *S. cerevisiae *and *S. pombe *plates in 384, 768 and 1536 format (see Fig. 7 for some example images that Colonyzer can deal with), amounting to several million quantified colony tile images. We typically execute large batches of analysis jobs on a 90 node Linux Beowulf cluster, but we can also analyze genome-wide screens within 36 hours by executing 8 simultaneous analysis jobs on a relatively inexpensive 64-bit dual quad-core Intel Xeon workstation with 12 Gb of RAM. By permanently archiving the source code on SourceForge [12,16] we hope that other groups and companies involved in high-throughput screening of micro-organism growth will use and develop this tool. Interested users, daunted by the prospect of installing many packages before trying Colonyzer, are welcome to email the corresponding author with an example image for analysis and can expect the return of Colonyzer output files to assess whether Colonyzer is useful for them.

On SourceForge we also provide a supplementary GUI tool to help users provide initial guesses for culture locations (Parametryzer), and code for summarising timecourse data with the logistic model (Logisticyzer). The latter also depends on our in-house Robot Object Database system (ROD) which will be released in the near future.

In future we are interested in improving the speed of analysis, while maintaining its sensitivity (which is its main feature), adapting Colonyzer to utilize some cloud computing services to achieve ever higher throughput, and to improve the ease with which users can install the packages required to run Colonyzer. Others may be interested in adding new functionality or disabling some Colonyzer functions to suit their particular requirements.

## Conclusions

This paper presents Colonyzer, an image analysis tool which specialises in quantification of cell density in micro-organism cultures growing on solid agar over a wide range of culture densities from plate photographs. Growing cultures on solid agar is often cheaper and less demanding than equivalent growth in liquid medium. Similarly, photographic image capture is cheaper and faster than spectrophotometric analysis of cell density in liquid wells. Colonyzer's particular strength is its sensitivity in detecting cultures with low density. It achieves sensitivity by correcting for any lighting gradients in captured photographs, and by segmenting images, differentiating between agar and culture, using a two-component Gaussian mixed model of pixel intensity. Algorithms underlying Colonyzer quantify densities of pinned cultures and all late-growth cultures well, but we have optimised them to tackle the more difficult problem of quantifying exponential-phase dilute liquid-inoculated culture density on agar. Colonyzer is significantly more accurate when compared to other tools in this regard. Colonyzer's sensitivity opens up the possibility of quantitative modelling of the growth curves of thousands of independent cultures grown on solid agar during high-throughput screens.

## Availability and requirements

**Project name**: Colonyzer

**Project home page**: http://research.ncl.ac.uk/colonyzer

**Operating system(s)**: Platform independent.

**Programming language**: Python & R

**Other requirements**: Python Imaging Library, RPy, NumPy, rgenoud

**License**: GNU GPL

**Any restrictions to use by non-academics**: No restrictions

## Authors' contributions

CL and DJW designed the algorithms, CL implemented them, wrote the manuscript and captured test images, AY captured test images and developed procedures for fitting the logistic model to Colonyzer output, SGA captured test images, evaluated Colonyzer quantifications, suggested algorithm improvements and contributed to the manuscript. DAL initiated the project, evaluated Colonyzer quantifications and contributed to the manuscript. All authors read and approved the final manuscript.
